# Nanotechnology-Based Diagnostic and Therapeutic Strategies for Neuroblastoma

**DOI:** 10.3389/fphar.2022.908713

**Published:** 2022-06-02

**Authors:** Hui Yan, Bo Zhai, Fang Yang, Zhenliang Chen, Qiang Zhou, Ana Cláudia Paiva-Santos, Ziqiao Yuan, Yang Zhou

**Affiliations:** ^1^ Children’s Hospital Affiliated to Zhengzhou University, Henan Children’s Hospital, Zhengzhou Children’s Hospital, Zhengzhou University, Zhengzhou, China; ^2^ Department of Cardiothoracic Surgery, Children’s Hospital Affiliated to Zhengzhou University, Henan Children’s Hospital, Zhengzhou Children’s Hospital, Zhengzhou, China; ^3^ Department of Pathology, Children’s Hospital Affiliated to Zhengzhou University, Henan Children’s Hospital, Zhengzhou Children’s Hospital, Zhengzhou, China; ^4^ Group of Pharmaceutical Technology, Faculty of Pharmacy, University of Coimbra, Coimbra, Portugal; ^5^ School of Pharmaceutical Sciences, Zhengzhou University, Zhengzhou, China

**Keywords:** neuroblastoma, nanotechnology, nanomedicines, diagnosis, therapy

## Abstract

Neuroblastoma (NB), as the most common extracranial solid tumor in childhood, is one of the critical culprits affecting children’s health. Given the heterogeneity and invisibility of NB tumors, the existing diagnostic and therapeutic approaches are inadequate and ineffective in early screening and prognostic improvement. With the rapid innovation and development of nanotechnology, nanomedicines have attracted widespread attention in the field of oncology research for their excellent physiological and chemical properties. In this review, we first explored the current common obstacles in the diagnosis and treatment of NB. Then we comprehensively summarized the advancements in nanotechnology-based multimodal synergistic diagnosis and treatment of NB and elucidate the underlying mechanisms. In addition, a discussion of the pending challenges in biocompatibility and toxicity of nanomedicine was conducted. Finally, we described the development and application status of nanomaterials against some of the recognized targets in the field of NB research, and pointed out prospects for nanomedicine-based precision diagnosis and therapy of NB.

## Introduction

### Epidemic Burden of Neuroblastoma

Neuroblastoma (NB), composed of undifferentiated neuroblasts, is an immature embryonal tumor originating from the adrenal medulla and paravertebral sympathetic nervous system (Matthay et al., 2016). The most common origin of NB is the adrenal gland, which occurs in 40% of localized tumors and 60% of metastatic tumors (Nakagawara et al., 2018). In addition, NB is also found to occur in other parts of the sympathetic nervous system other than the adrenal gland (Pudela et al., 2020). As the most common cancer in infancy and the most common extracranial solid tumor in childhood, NB is the third most frequent childhood tumor, ranking after leukemia and brain tumors and accounting for 6–10% of pediatric tumors (Nicholas et al., 2016). Some high prevalence countries such as France, Israel, Switzerland and New Zealand have an annual incidence of 11/1 million (0–15 years), the United States has about 25/1 million, and China and India have less than 5/1 million (Moreno et al., 2016). The incidence of NB is age-dependent, with a mean age of 17.3 months at the time of clinical diagnosis (Matthay et al., 2016; Whittle et al., 2017). It has been estimated that NB accounts for 9–15% of all childhood cancer-related deaths and is a highly heterogeneous tumor, with 5-year survival rates of 90 and 50% for patients with NB in the non-high-risk and high-risk groups, respectively (Pudela et al., 2020).

### Difficulty in Neuroblastoma Diagnosis

NB is diagnosed on the basis of histological confirmation combined with clinical manifestations, laboratory tests, imaging features and genetic examinations (Zhan et al., 2017; Swift et al., 2018a). Imaging analysis as visualization detection methods are irreplaceable in NB diagnosis and mainly include ultrasound imaging (US), magnetic resonance imaging (MRI), and optical imaging. However, the pathological characteristics of most tumors usually cannot be accurately estimated by imaging analysis alone. Ancillary detections of some specific tumor biomarkers such as neuron-specific enolase, S-100 protein and tryptophan were performed to improve the histological diagnostic accuracy of NB (Rajwanshi et al., 2009). In about 90% of NB cases, increased catecholamines and their metabolites includes dopamine, homovanillic acid and vanillylmandelic acid are found in the urine or blood (Weinstein et al., 2003). Also, the combination of tissue biopsy and pathological examination is an indicator of a definitive diagnosis of the disease. Actually, a synthetic assessment based on histological verification combined with chemical analysis and imaging features is necessary for the diagnosis of NB for a comprehensive assessment of disease progression (Swift et al., 2018b). Since biological methods have limited sensitivity and specificity and failure in precise tumor localization, invasiveness, and limited specimen acquisition, the development of imaging techniques will more likely to achieve early diagnosis of NB (Swift et al., 2018a). However, imaging methods are constrained by cost and risk, while it remains powerless for tumors smaller than 0.5 cm in diameter, thus it is urgent to develop advanced techniques for early diagnosis and monitoring of NB.

### Current Challenges in Neuroblastoma Treatment

Although complete resection of the primary NB is expected to greatly improve overall survival and most children are inoperable due to metastases at the time of diagnosis, thus chemotherapy, radiotherapy, differentiation-inducing therapy, immunotherapy, and autologous hematopoietic stem cell transplantation remain the primary treatments in most cases (Perkins et al., 2014; Berlanga et al., 2017; Sait and Modak, 2017). The chemotherapeutics clinically used for NB include cisplatin, cyclophosphamide, vincristine, etoposide, teniposide, Adriamycin (DOX) (Berlanga et al., 2017). As a method of local treatment of tumors, nuclear medicine treatment is suitable for controlling localized tumors that cannot be completely removed or with unsatisfactory effect of chemotherapy (Perkins et al., 2014). Currently, the most promising immunotherapy is monoclonal antibody technology against the ganglioside 2 (GD2), and has achieved remarkable therapeutic results in the consolidation phase (Sait and Modak, 2017). Autologous stem cell transplantation is also applied greatly in clinical NB treatment with the advantages of low self-recurrence rate, early recovery from immune reconstitution, and rapid recovery from bone marrow transplantation (Weinstein et al., 2003). Conventional treatment has been shown to achieve good results in children with low-risk NB, but the outcome in children with high-risk NB remains unsatisfactory even with various combined treatments. Nevertheless, there are some insurmountable limitations of conventional therapies that require further improvement. Surgical intervention may result in incomplete tumor resection. Chemotherapy is highly prone to damage healthy tissues, leading to severe, dose-limiting side effects, including toxicity and bone marrow suppression, compromising efficacy and even leading to chemoresistance. In addition, emerging targeted drugs exhibit many drawbacks, such as high toxicities, low cure rates, and high off-target propensity (Hishiki et al., 2014).New treatment concepts are urgently needed to effectively treat children with NB, and nanomedicine as an emerging technology could provide better personalized treatment for tumor patients.

### Prospects of Nanotheranostics for Precise Diagnosis and Treatment in Neuroblastoma

**FIGURE 1:**
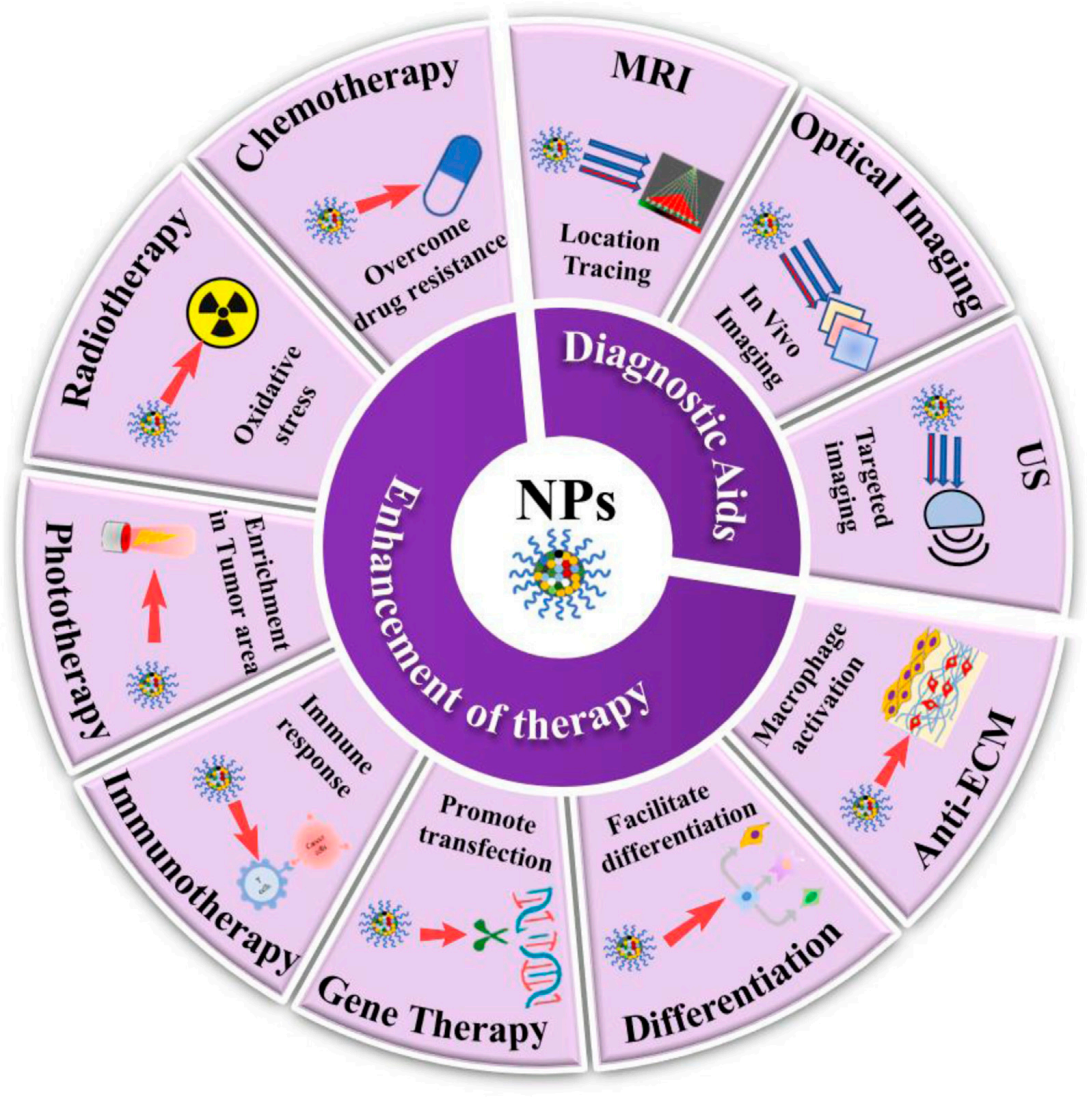
Schematic illustration of emerging nanomedicines for NB diagnosis (MRI, optical. imaging and US) and therapies (chemotherapy, radiotherapy, phototherapy, immunotherapy, gene therapy, differentiation, and anti-ECM therapy).

Nanomedicine refers to nanoparticles (NPs) whose size range is 1–1000 nm in pharmacy. Nanocarriers are various nanomaterials capable of dissolving or dispersing drugs, while nanodrugs are NPs processed directly from active pharmaceutical ingredients (Sosnik and Carcaboso, 2014; Raza et al., 2019). The main types of NPs include nanoliposomes, nanocapsules, nanospheres, solid lipid NPs, polymer micelles, and nanomedicines (Aleassa et al., 2015). The contrast agent encapsulated by nanomaterials facilitate the acquisition of detailed cellular and molecular images, real-time detection of targeted drugs within the tumor, and also provides more detailed data for maximum tumor removal, thus improving the diagnostic accuracy (Aleassa et al., 2015; Wang et al., 2022a). Nevertheless, the application of nanotechnology in NB therapy remains a challenging new strategic attempt. Nanodrug delivery systems possess superior advantages over conventional means in overcoming limitations associated with unfavorable drug properties, such as solubility, stability, permeability, toxicity, and increased drug accumulation in desired tumor-specific areas and thus eliminating unwanted side effects and toxicity (Rodríguez-Nogales et al., 2019; Raza et al., 2022). Nanodrug technology will be an emerging and more promising therapeutic strategy in the field of NB treatment, especially for high-risk NB patients who have suffered from failure in conventional treatment or relapsed. Here, we are summarizing the current literature on nanotechnology and providing insights into the applications of nanotechnology-based diagnosis and therapeutic strategy for NB, including US, MRI, optical imaging, chemotherapy, radiation therapy, phototherapy, immunotherapy, gene therapy, differentiation and tumor extracellular matrix (ECM) remodeling (Figure 1). Moreover, the opportunities and challenges of nanomedicine in the field of oncology research are described in detail, especially the biocompatibility and toxic effects of nanomaterials. Finally, some recognized targets for NB diagnosis or treatment are also highlighted, as well as a special focus on the current status of nanotechnology development and applications based on them.

## Nanotechnology Enables Neuroblastoma Diagnosis

Although image analysis is irreplaceable as a visualization detection method in NB diagnosis, it has the drawbacks of low detection capability and low accuracy rate. The development of nano-contrast agents or nanoprobes suitable for various types of imaging devices are currently available to visualize the physiological, pathological and metabolic conditions of the body with high accuracy and will present prospects for application in the diagnostic and prognostic evaluation of NB. This section focused on recently proposed approaches for the diagnosis of nanomedicines in patients with NB (Table 1).

**TABLE 1:** Summary of emerging nanomedicines for NB diagnostics.

Diagnostic type	Formulation	Type of NPs	Sizes (nm)	Model	Observed effects	Ref.
US	RVG-GNPs	Nanobubbles NPs	220	*In vitro* and *in vivo*	Enhanced US signals and reduced tumor growth in a tumor-bearing mouse model.	Lee et al. (2016a)
MRI	DySiO_2_-(Fe_3_O_4_)_n_	Inorganic NPs	30	*In vitro*	High-performance MRI and fluorescence imaging of NB	Lee et al. (2006b)
	Fe3O4-poly(acrylic acid) (PAA)	Magnetic polymer particles	20–200	*In vitro* and *in vivo*	Exhibited highly biocompatible and good contrast in T2-weighted imaging.	Chen et al. (2015)
	Fe3O4@GdPB	Iron oxide-gadolinium-containing Prussian blue	About 30	*In vitro* and *in vivo*	Increased the signal: noise ratio of the T1-weighted scan and reduced the growth rate of the tumor.	Kale et al. (2017)
	LPD	Composite of liposome, peptide and plasmid DNA	70–140	*In vitro* and *in vivo*	Targeted NB cell transfection and real-time monitoring of vector distribution in the tumor	Kenny et al. (2012)
Optical imaging	NDI-nip FONPs	Organic particles	50–70	*In vitro*	Targeted imaging and delivery of curcumin to NB cell	Ghosh et al. (2021)
	A&MMP@Ag2S-AF7P	Affinity peptide composites	160	*In vitro* and *in vivo*	Distinguish tumor tissue from non-cancerous tissue	Zhan et al. (2021)
	Anti-GD2/GQDs	Conjugates of graphene quantum dots and antibody	150–160	*In vitro* and *in vivo*	Tumor tracking and imaging	Lin et al. (2021)

### Nanotechnology for US

US is unique in the field of medical imaging due to its safety and convenience and is usually performed for the first examination when an abdominal mass is suspected in a child (Swift et al., 2018b). In contrast to other types of diagnostic methods such as x-ray and computed tomography, US performs deeper tissue penetration and less invasiveness to the organism. Modification of contrast agents for US is one of the main directions regarding the improvement of ultrasound diagnostic performance. Through the integration with multiple nanosystems, ultrasound not only enables better high-resolution ultrasound imaging, but also facilitates the controlled release of drugs at specific tumor sites (Alphandéry, 2022). Although there have been numerous studies on the application of nanomaterial-based modified ultrasound technology for cancer diagnosis, i.e., nano-bubbles and inorganic NPs, there are still few reports on the diagnosis of NB (Devarakonda et al., 2017; Liu et al., 2019). Interestingly, Lee Jet al. have created cancer-targeting, gas-generating polymer NPs (GNPs) as a therapeutic tool for ultrasound imaging and treatment of NB (Lee et al., 2016a). In detail, the composite of GNPs modified with rabies virus glycoprotein (RVG) peptides that specifically target NB cells (RVG-GNPs) was used as the nanoimaging material. Importantly, it was found to greatly enhance the ultrasound signal in a tumor-bearing mouse model and to suppress tumor growth without conventional therapeutic agents. Since children are a vulnerable population to toxic contrast and therapeutic agents, adopting this approach promisingly enables both targeted therapy and highly accurate detection with guaranteed safety.

### Nanotechnology Facilitates Magnetic Resonance Imaging

**FIGURE 2:**
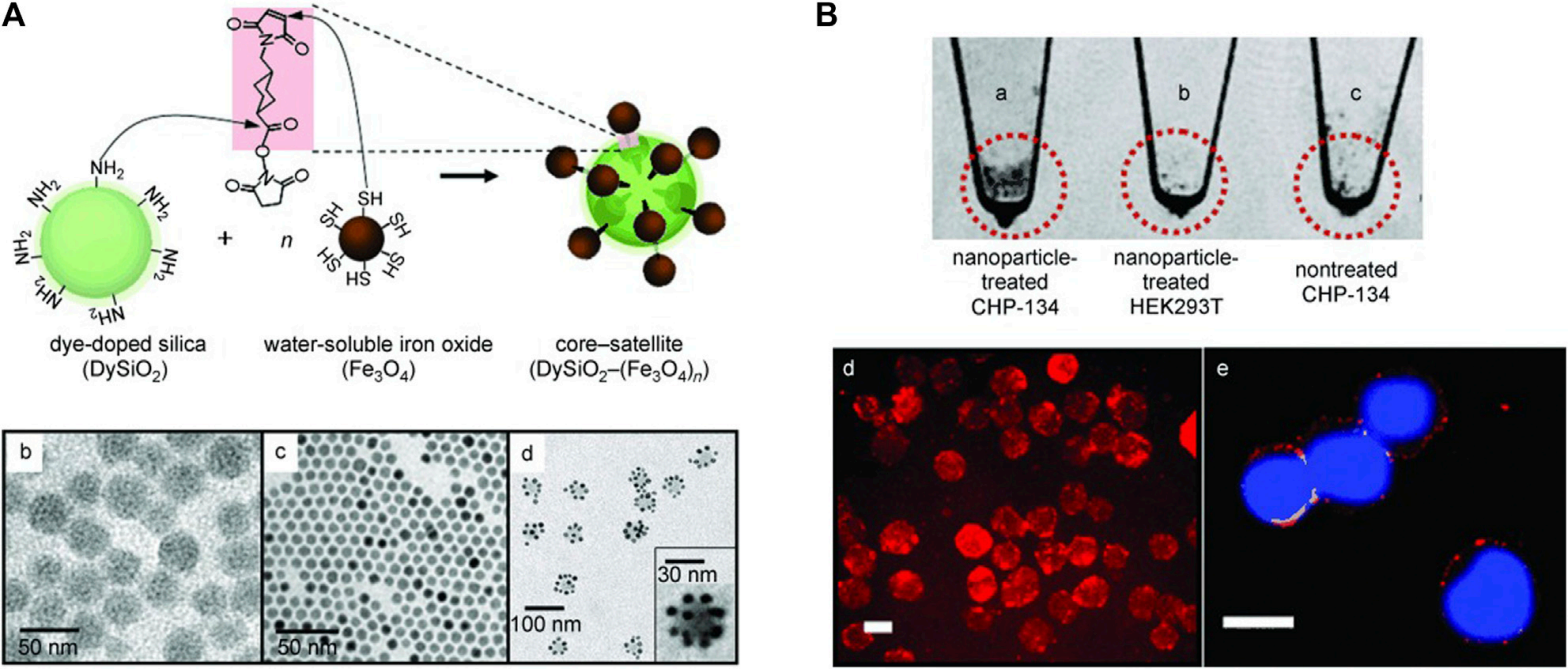
Dual-Mode nanoparticle probes for high-performance magnetic resonance and fluorescence imaging of NB. **(A)** Schematic diagram for the synthesis of core–satellite DySiO2–(Fe3O4)n nanoparticles. **(B)** Dual-mode detection of PSAs. Reproduced with permission from (Lee et al., 2006a). Copyright 2006, Wiley-VCH Verlag GmbH & Co. KGaA, Weinheim.

Magnetic NPs (MNPs) are extremely promising for NB imaging and targeted therapies. Modification of peptides or antibodies on the surface of MNPs allows direct targeting of tumor cells to disrupt the function of tumor cell-active signaling pathways (Chertok et al., 2008). MNPs show higher longitudinal relaxation, present an enhanced signal on T1-weighted images and shorten the transverse relaxation time in T1-and T2-weighted images, resulting in a significant decrease in signal intensity of the target organ on conventional T2-weighted images. Therefore, MNPs have been widely used as contrast agents and molecular imaging probes for MRI. Hybrid nanoparticle probes fabricated using rhodamine-dy-doped silica (DySiO2) NPs as the core material conjugated with high-quality water-soluble Fe3O4 (WSIO) NPs have been confirmed to have synergistic MRI enhancement and good fluorescence properties for polysialic acids (PSAs)-expressing NB cells (Figure 2) (Lee et al., 2006a). Superparamagnetic Fe3O4 NPs encapsulated in porous polyacrylic acid (PAA) nanogels to make hybrid nanodrugs with high drug loading capacity, good contrast in T2-weighted imaging, and high MRI sensitivity at NB cell-derived tumor sites (Chen et al., 2015). Composite iron oxide-gadolinium-containing Prussian blue NPs (Fe3O4@GdPB) perform a dual function as a novel therapeutic nanoparticle for diagnostic and therapeutic purposes, which is manifested in the availability for T1-weighted MRI and photothermal therapy (PTT) in NB cell-derived mouse tumor model (Kale et al., 2017). Notably, Fe3O4@GdPB NPs acted as effective MRI contrast agents and were able to effectively improve the signal-to-noise ratio of T1-weighted scans of tumor *in vivo*. Another study reported that a nanocomplex named as LPD, which composed of cationic DOTMA/DOPE liposomes (L), neurotensin-targeting peptide (P) and plasmid DNA (D) fulfilled the multiple functions of both targeted NB cell transfection and real-time monitoring of vector distribution in the tumor by MRI (Kenny et al., 2012).

### Nanotechnology Adjunct to Optical Imaging

**FIGURE 3:**
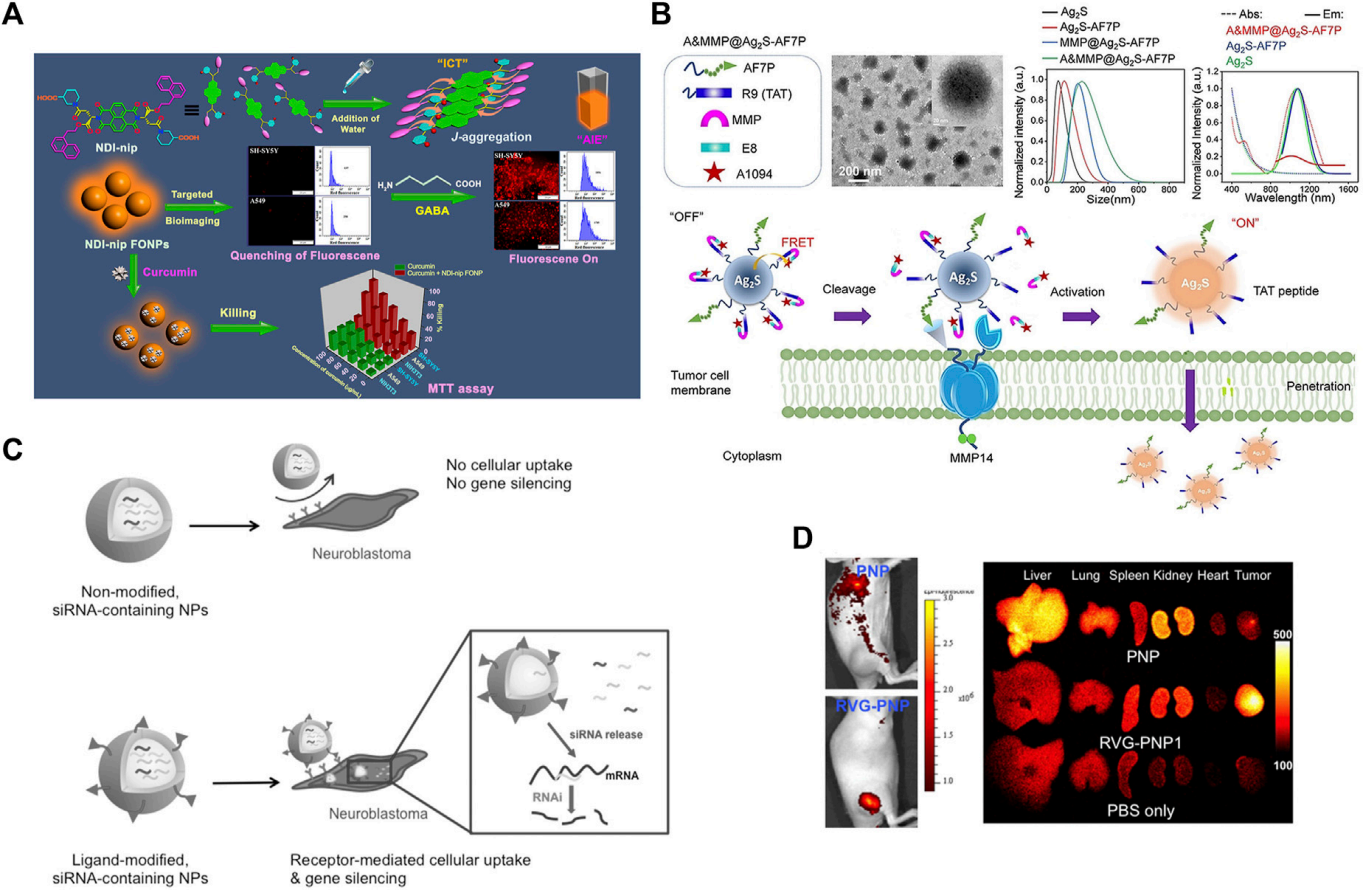
Nanoparticle for optical imaging of NB. **(A)** Schematic diagram of targeted cell imaging of GABA (γ-aminobutyric acid)-rich SH-SY5Y cells by NDI-derived FONPs. Reproduced with permission from: Anup Kumar Ghosh, Monalisa Chowdhury, and Prasanta Kumar Das. Nanoparticles as Targeted Delivery Vehicle and Diagnostic Probe toward GABAA-Receptor-Enriched Cancer Cells. ACS applied bio materials, 2021, 4(10): 7563–7577 (Ghosh et al., 2021). Copyright 2021, American Chemical Society. **(B)** Schematic illustration of A&MMP@Ag2S-AF7P for NB detection. Reproduced with permission from (Zhan et al., 2021). Copyright 2020, Wiley-VCH Verlag GmbH & Co. KGaA, Weinheim. **(C–D)** Ligand-modified, gene-loaded NPs as a tumor-targeting theranostic agent. Reproduced with permission from (Lee et al., 2016b). Copyright 2015, Wiley-VCH Verlag GmbH & Co. KGaA, Weinheim.

Although optical imaging is non-radioactive, non-invasive, high-resolution and controllable, its penetrating power is relatively inferior. Fluorescence imaging based on fluorescence signals generated by fluorescein compensates for this deficiency to some extent. Near-infrared fluorescence (NIRF) probes are widely adopted for their high transmission capability and safety, and have been implemented in the field of small animal bioimaging systems and translational medicine research on tumors. Nowadays, there are numerous nanomaterials such as liposomes, metallic as well as nonmetallic NPs are available to encapsulate NIRF for targeted tumor optical imaging (Setua et al., 2010). Fluorescent organic NPs (FONPs) derived from naphthalenediimine (NDI) can be used as targeted diagnostic probes for targeted cellular imaging and as drug delivery vehicles for the delivery of the anticancer drug curcumin to γ-aminobutyric acid receptor-rich cells such as the NB cell line SH-SY5Y (Figure 3A) (Ghosh et al., 2021). Such spherical organic particles are formed by self-assembly driven by piperidine-tethered l-aspartate attached NDI derivatives occurring through J-type aggregation, exhibiting aggregation-induced emission. It has been proposed that a matrix metalloproteinase 14 (MMP14)-activatable NIR-II nanoprobe (A&MMP@Ag2S-AF7P) can be used to distinguish the NB tumor tissues from surrounding non-cancerous tissue (Figure 3B) (Zhan et al., 2021). This nanoprobe consists of three main functional components, an affinity peptide AF7P targeting the membrane-type ring structure of MMP14, an MMP14 activating peptide, and a fluorescence resonance energy transfer (FRET) system with NIR-II-emitting Ag2S QDs and a NIR absorber A1094. Cooperative interaction between its components selectively produces visible fluorescent signals in NB tissues with high expression of MMP14, which facilitates rapid and unperturbed tissue analysis for *ex vivo* NB diagnosis and greatly quickens intraoperative decisions. Another study reported that utilizing the nanomaterial graphene quantum dots (GQD) coupled with anti-GD2 antibodies enables tumor tracking and imaging in mice with minimal or no *in vitro* cytotoxicity (Lin et al., 2021). In addition, a biocompatible poly(d,l-lactide-co-glycolide) (PLG) nanoparticle containing imaging probes and therapeutic genes modified with RVG peptide can effectively target NB tumor sites for optical imaging both *in vitro* and *in vivo*, and can carry nanoparticles encapsulating therapeutic genes (siMyc, siBcl-2, and siVEGF) to significantly inhibit tumor growth in mouse models for targeted therapeutic effects (Figures 3C,D) (Lee et al., 2016b). Multimodal imaging technology based on nanoimaging agents integrates various types of imaging modalities to produce synergistic effects and provide more comprehensive and accurate imaging information for precise diagnosis and treatment of NB.

## Nanotechnology Enhances Therapeutic Treatment for Neuroblastoma

### Nanotechnology and Chemotherapy

Chemotherapy, as a conventional therapeutic strategy for NB, has yielded great achievements in narrowing tumor area prior to surgery resection, preventing tumor metastatic spread, suppressing tumor proliferation, and prolonging patients' lifetime. Chemotherapeutics routinely administered for NB include several cytotoxic agents such as vincristine, DOX, cyclophosphamide, cisplatin, carboplatin, topotecan, irinotecan, and paclitaxel (PTX) (George et al., 2010). However, curative effects of these chemotherapeutic agents tended to be severely compromised due to their rapid clearance and non-specific distribution, which leads to unavoidable systemic toxicity. In addition, multidrug resistance is another major cause of chemotherapy failure (Wu et al., 2022). Currently, nanomaterial-based approaches have been proposed in combination with chemotherapy aim to enhance the efficacy of conventional chemotherapy regimens through multiple strategies. Nanomedicines enhance the effectiveness of chemotherapy for NB mainly through the following ways: 1) targeting chemotherapeutics through nanocarriers; 2) improving permeability to tumor tissues; 3) reversing multiple drug resistance; 4) collaborating with other therapeutic approaches for NB.

The abnormal vascular proliferation in tumor tissues is characterized by high vascular density and poor vessel wall integrity, wide gaps, permeability to macromolecular particles and comparatively slow lymphatic reflux, thus enabling NPs of a certain size to access and retain in tumor tissues, and achieving efficient and accurate enrichment in tumor tissues. Such effect is called the enhanced permeability and retention (EPR) effect, which belongs to the passive targeting effect (Matsumura and Maeda, 1986). Besides, various nanomaterial-based targeted drug delivery systems have been designed to enhance the aqueous solubility, stability and pharmacokinetics process of numerous hydrophobic drugs *in vivo*, enabling aggressive targeting of drug delivery to tumor sites, realizing targeted drug release and reducing drug toxicity while enhancing drug efficacy and overcoming drug resistance. NPs with diameter less than 200 nm exhibit stronger EPR efficacy which are widely utilized for tumor targeting therapy. Nanomaterials have a large specific surface area and are able to effectively load hydrophobic drugs, exhibit protective effect and increase their stability and bioavailability in the circulation, which allows for long circulation through modifications such as polyethylene glycol (PEG) (Wen et al., 2012). The small molecule chemotherapeutic agents mainly penetrate into the tumor cells through passive diffusion, which is less efficient and poorly targeted, susceptible to drug resistance through recognition and efflux by the transporter proteins on the membrane surface of tumor cells. Overcoming drug resistance by loading chemotherapeutics with nanomaterials to alter the drug delivery to tumor cells and improve drug uptake by tumor cells is considered to be a feasible and promising strategy (Khan et al., 2019; Amerigos Daddy J C et al., 2020). It was found that liposomal NPs overcame drug resistance by mediating the entrance of drugs into tumor cells through cytokinesis and further evading the efflux effect of transporter proteins and lysosomal phagocytosis (Li et al., 2013). Several cationic polymers such as Planic are characterized by their transportation to the nucleus (Batrakova and Kabanov, 2008), and such materials would be utilized for the loading of chemotherapeutics that specifically target the nucleus to allow them to deliver smoothly to the intended target.

Research reveals that NPs carriers may provide better anti-tumor efficacy of chemotherapeutics, especially topoisomerase inhibitors and PTX, in NB (Table 2). SN-38 as a novel topoisomerase I inhibitor exhibits an extensive anticancer activity in adult and pediatric tumors (Norris et al., 2014). However, this camptothecin (CPT) analogue is limited in clinical use due to its toxicity, metabolic instability and incompatibility with conventional drug delivery vehicles (Colletti et al., 2017). EZN-2208, a water-soluble polyethylene glycolized SN38 drug coupling, possesses strong cytotoxicity to NB cells, prolongs duration of drug activity, and is well tolerated *in vivo* without significant toxicity, acute or chronic hepatic and renal toxicity. The employment of hydroxypropyl-β-cyclodextrin (HP-β-CD) derivatives as a drug delivery system significantly enhanced the stability, bioactivity and antitumor activity of the alkaloids CPT and luotonin A in several cancer cell lines including breast, lung, liver, ovarian and NB (González-Ruiz et al., 2021). To improve the solubility and pharmacokinetic properties of chemotherapeutics such as cisplatin, and azithromycin, albumin NPs and liposomes were developed and exhibited strong inhibitory activity against NB cell line (Vignaroli et al., 2016). Mulik et al. produced apolipoprotein-E3-mediated curcumin-loaded poly(butyl) cyanoacrylate NPs using an anionic polymerization method (Mulik et al., 2012), and the formulation showed enhanced anticancer activity against SH-SY5Y cell compared to the natural curcumin solution and untargeted NPs. Albumin-bound PTX NPs (Nab-PTX) exhibited enhanced cellular transport capacity, better cytotoxic effect in NB cells-derived mouse model compared to non-encapsulated PTX (Zhang et al., 2013). Similarly, the nano-encapsulation formed by the biocompatible drug delivery vehicle Poly (lactic-co-glycolic acid) (PLGA) with PTX exhibited stronger cytotoxic effects on NB cells than that of free PTX (Bacanlı et al., 2021). Excitingly, the safety and efficacy of Nab-PTX has been assessed in Phase I/II clinical trials for refractory NB and other pediatric solid tumors (NCT01962103) (Moreno et al., 2018; Amoroso et al., 2020). Although limited activity was observed, the safety of Nab-PTX was confirmed in pediatric patients. Above all, incentivizing the effective delivery of drugs and improving their bioavailability and efficacy *in vivo* are the critical issues that should be addressed in a wide range of tumor treatments, including NB.

**TABLE 2:** Summary of emerging nanomedicines for NB chemotherapy.

Formulation	Type of NPs	Drug	Model	Observed effects	Ref.
CPT/HP-β-CD and CPT/β-CD	Cyclodextrin	CPT	*In vitro*	Enhanced the bioactivity and antitumor activity of the CPT	González-Ruiz et al. (2021)
LP-2- pyrazolo[3,4-d]pyrimidines	Liposome	Pyrazolo[3,4-d]pyrimidines	*In vitro*	Exhibited inhibitory activity against NB cell	Vignaroli et al. (2016)
ApoE3-C-PBCA- Curcumin	ApoE3-C-PBCA	Curcumin	*In vitro*	Enhanced anticancer activity of curcumin against NB cell	Mulik et al. (2012)
Nab-PTX	Nab	PTX	*In vitro* and *in vivo*	Inhibited NB cells growth and prolongs the survival of tumor-bearing mice	Zhang et al. (2013)
PLGA-PTX	PLGA	PTX	*In vitro*	Induced DNA damage in NB cells	Bacanlı et al. (2021)
Nab-PTX	Nab	PTX	phase I/II	Relatively safe for children with NB	(Moreno et al., 2018; Amoroso et al., 2020)

### Nanotechnology and Radiotherapy

**FIGURE 4:**
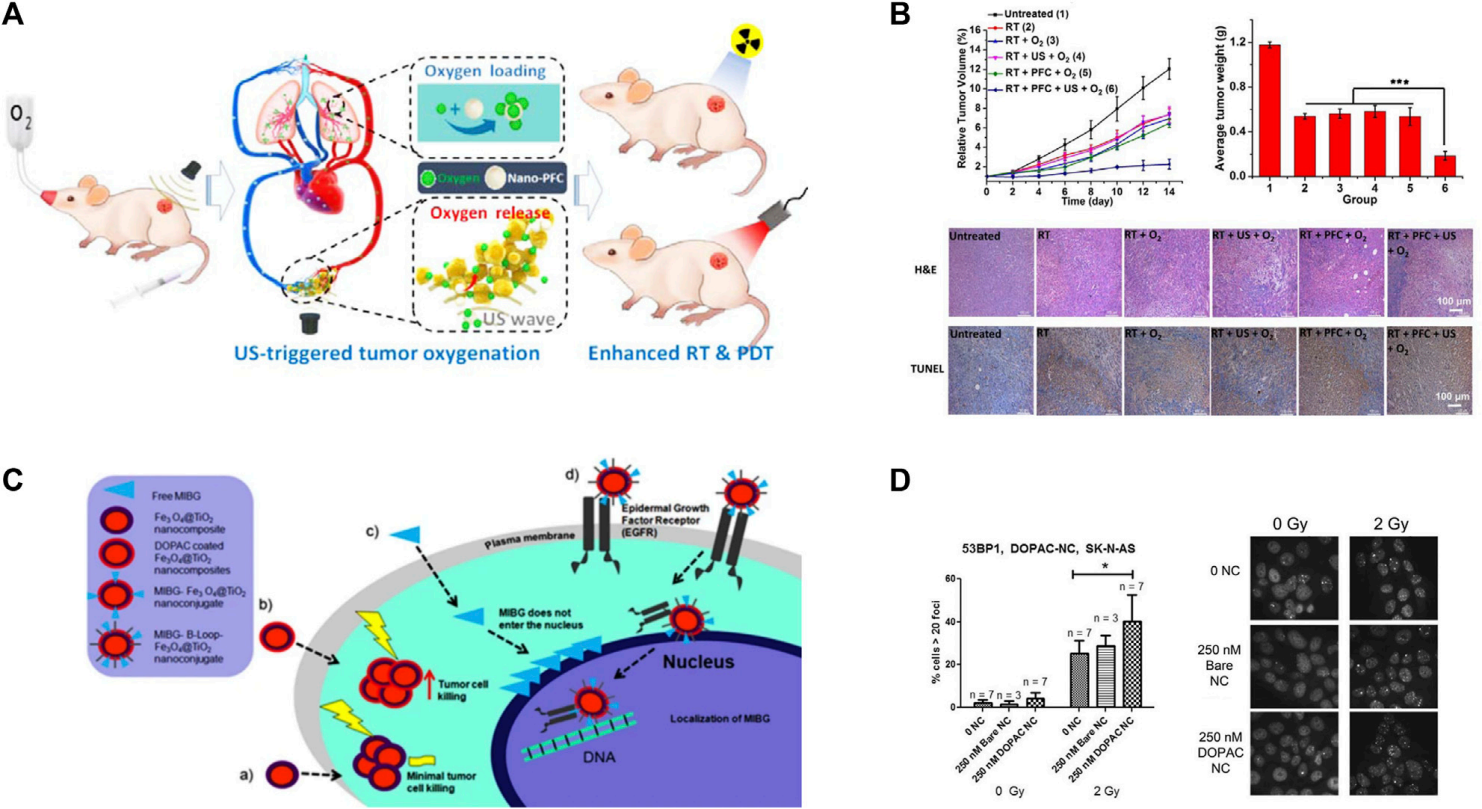
Nanotechnology enhances radiotherapy in NB. **(A–B)** The tumor hypoxic microenvironment was modulated using perfluorocarbon nanoparticles as an oxygen shuttle, which significantly improved the therapeutic effect of PDT and RT on tumors. Reproduced with permission from: Xuejiao Song, Liangzhu Feng, Chao Liang, Kai Yang, and Zhuang Liu. Ultrasound triggered tumor oxygenation with oxygen-shuttle nanoperfluorocarbon to overcome hypoxia-associated resistance in cancer therapies. Nano letters, 2016, 16(10): 6145–6153 (Song et al., 2016). Copyright 2016, American Chemical Society. **(C–D)** Schematic diagram of MIBG and DOPAC-coated Fe3O4@TiO2 as potential radiosensitizers for NB. Reproduced with permission (Liu et al., 2021). Copyright 2021, Springer Nature.

Although radiotherapy is one of the standard therapies applied for NB, its therapeutic efficacy is not satisfactory due to adverse effects, radioresistance and recurrence after radiotherapy. Upon radiation photon incidence, metal nanomaterials with higher atomic number (such as metal atoms of gold and silver) undergo energy level jumps and release oscillating electrons, simultaneously scattering Compton electrons (Bilynsky et al., 2022). The high density of ionization energy generated by these electrons on the surface of NPs leads to enhanced radiation energy and enrichment in tumor cells, which strengthens the radioactive DNA damage and further facilitates DNA double-strand breaks, suppresses DNA synthesis and repair (Nasir et al., 2021). Meanwhile, such ionization increases the production of free radicals in tumor cells and enhances the lethality of tumor cells (Calugaru et al., 2015). Furthermore, therapeutic effects of radiotherapy can be further improved with functional nanomaterials through modifying tumor hypoxia. Hemoglobin-based nanocarriers and perfluorocarbon-based NPs have been confirmed to facilitate the EPR effect of NPs via oxygen molecule loading on nanocarriers in preclinical animal experiments, thus efficiently enhancing the tumor oxygen levels, averting the hypoxic tumor microenvironment, and sensitizing tumor to radiotherapy (Figures 4A,B) (Murayama et al., 2012; Song et al., 2016). It has also been reported that MnO2 nanomaterials represent a promising nanomaterial for radiotherapy sensitization (Gong et al., 2018). William et al. has found that exposed m-iodobenzylguanidine (MIBG) and 3,4-dihydroxyphenylacetic acid (DOPAC) coated Fe3O4@TiO2 nanocomposites significantly increased the sensitivity of NB cells to radiotherapy (Figures 4C,D) (Liu et al., 2021). In addition, this nanocomposite also partially penetrates into the nucleus through mimicking the presence of epidermal growth factor peptides, thus enabling targeting of radiolabeled MIBG molecules into the nucleus with nucleus-targeting nanostructures in future. The nanocoupler can be used as a radiosensitizer for external irradiation therapy and also for delivery of internal emitters to near-genomic DNA regions.

### Nanotechnology and Phototherapy

In recent years, phototherapy has received increasing attention in NB treatment in view of its great advantages in improving oncologic outcomes and diminishing side effects. Phototherapy mainly includes two types of therapies, photothermal therapy (PTT) and photodynamic therapy (PDT). Given the intrinsic characteristics of NB tumor tissues with tortuous blood vessels and low heat dissipation efficiency when undergoing heat, PTT is capable to selectively destroy tumor cells while preventing damage to normal cells. Although PTT is relatively safe and controllable, it is less permeable to deep tissues and has limited heat enrichment. Nanomaterials in PTT inherently possess therapeutic properties. Light absorbers typically include nanogold, graphene, and NIR dyes (Zakaria et al., 2016). Nanomaterials with photothermal effect that can effectively convert light energy into thermal energy specifically in tumor cells will undoubtedly beneficial in minimizing damage to surrounding tissue.

The photosensitizers used for PDT-based tumor suppression mainly have two types: type I process in which the photosensitizer reacts directly with components of the cellular microenvironment to produce peroxides or superoxides that oxidatively disrupt tumor cells; and type II process in which a porphyrin-containing photosensitizer is utilized to produce highly reactive singlet oxygen and thus kill tumor cells upon light exposure at specific wavelengths (Tian et al., 2016). The ROS generated by the photosensitizer have short half-life and the cells adjacent to the photosensitizer undergo the PDT process following photosensitizer enrichment into the tumor area through nanomaterials, thus effectively reducing damage to surrounding normal tissues (Bechet et al., 2008). Photosensitizers themselves tend to be off-target, poorly water-soluble and bioavailable, and developing photosensitizers using nanomaterials will be a superior approach to overcome these flaws (Lim et al., 2013).

**FIGURE 5:**
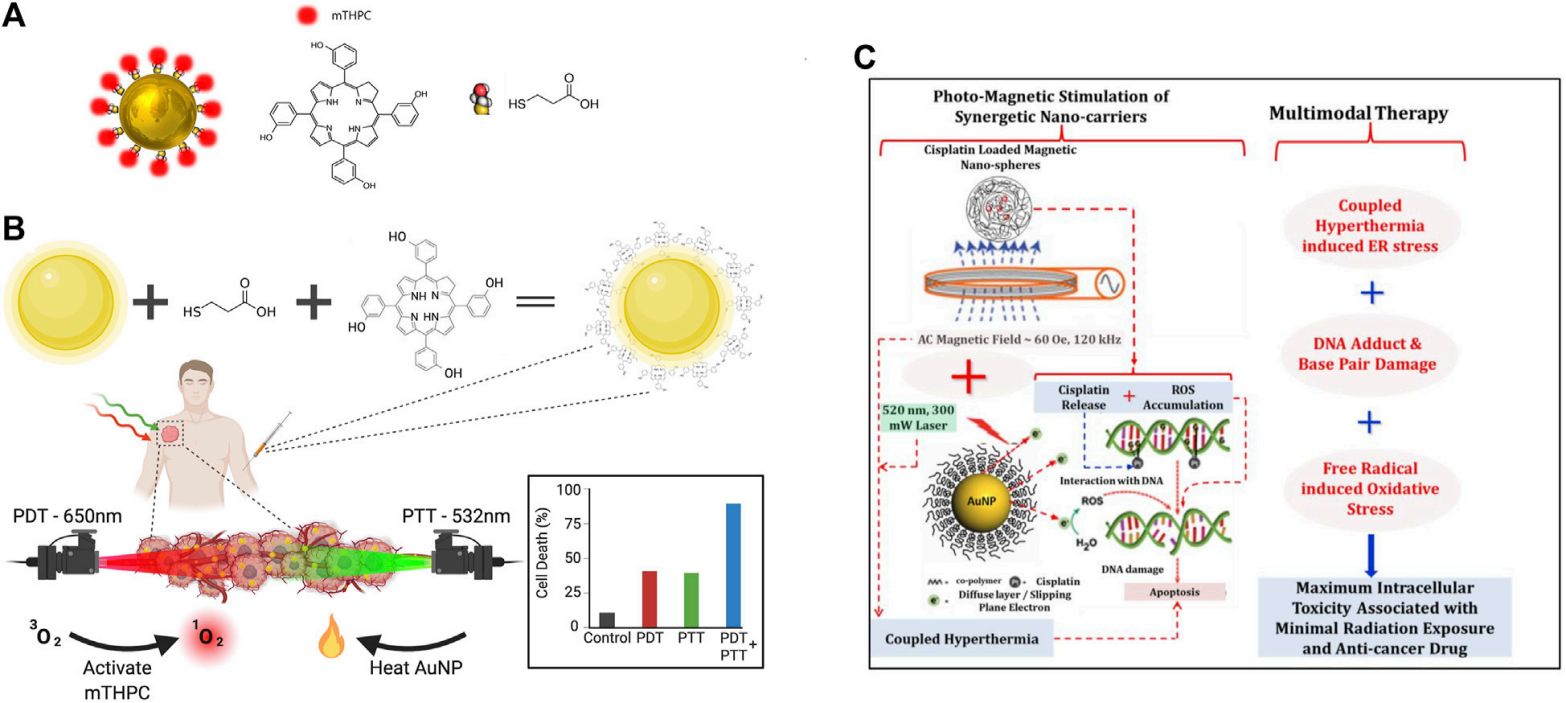
Nanotechnology for phototherapy in NB. **(A)** The AuNP-mTHPC complex is illustrated with the chemical structure of mTHPC in the conjugate and the linker 3-mercaptopropionic acid. Reproduced with permission from: Elina Haimov, Hana Weitman, Shlomi Polani, Hadas Schori, David Zitoun, and Orit Shefi. meso-tetrahydroxyphenylchlorin-conjugated gold nanoparticles as a tool to Improve Photodynamic Therapy. ACS applied materials & interfaces, 2018, 10(3): 2319–2327 (Haimov et al., 2018). Copyright 2018, American Chemical Society. **(B)** AuNP-mTHPC complexes for PDT and PTT dual therapy. Reproduced from (Varon et al., 2022). **(C)** Photo-magnetic irradiation mediated multimodal therapeutic strategy of NB cells. Reproduced from (Atluri et al., 2018).

Complex iron oxide-gadolinium-containing Prussian blue NPs (Fe3O4 @GdPB) have become novel and effective PTT nanotherapeutic agents for reducing tumor growth rate and improving survival, owing to its cytotoxic effect on targeted tumor cells exposed to laser irradiation (Kale et al., 2017). NIR dyes and porphyrin analogs afford both multimodal imaging and PTT effects. For example, hyaluronic acid-anthocyanine-like dye-iron composite NPs are available for fluorescence imaging, magnetic resonance multimodality imaging and PTT (Della Sala et al., 2022; Wang et al., 2020; Hu et al., 2015). It is rapidly elevated to 28°C with approximately 5 min of irradiation at 1 W/cm2 under a 785 nm laser to facilitate tumor elimination in MCF-7 xenograft mice (Tian et al., 2016). Recently, the dual application of PDT and PTT for better therapeutic outcomes in cancer treatment has attracted a great deal of interest. Gold NPs (AuNP) are well suited as drug carriers due to their biocompatibility, ease of fabrication, and multiple physical properties, making them highly desirable for combining multiple therapeutic approaches in a variety of tumor treatments (Figure 5A) (Haimov et al., 2018). Based on this, a group has synthesized a functional complex based on AuNPs covalently linked to meso-tetrahydrobenzyl chloride (mTHPC) drug molecule (AuNP-mTHPC), which enhances the pro-cell death effect of PDT in NB (Haimov et al., 2018). Subsequently, they found that this highly biocompatible and soluble nanotherapeutic agent additionally exhibits a dual PDT/PTT phototoxic effect on NB cells (Figure 5B) (Varon et al., 2022).

In addition, the combination of mesoporous carbon NPs-based nanoformulations with chemotherapeutic agents has been found to trigger synergistic tumor suppressive effects with PDT or PTT therapy. For example, combining doxorubicin with mesoporous carbon NPs significantly improved the inhibitory effect of both PTT and PDT on tumor cells (Bechet et al., 2008). However, photothermal nanomaterials, especially inorganic nanomaterials, are not easily degradable in organisms and possess potential toxicity as well as the immune-related adverse effects. To reduce the intensity of photomagnetic irradiation and the required nanoparticle dose level, a hybrid photomagnetic irradiation method based on intelligent nanostructures were developed and exhibited high efficiency and low toxicity in killing NB cells (Figure 5C) (Atluri et al., 2018). Development of photothermal conversion materials with greater biocompatibility, degradability and lower toxicity will be a hot spot for future phototherapy.

### Nanotechnology and Immune-Based Therapy

Immunotherapy as an emerging anti-cancer therapy refers to the comprehensive homeostatic regulation of tumor suppression through reactivation of the immune function or removal of immunosuppression in the organism (Raza et al., 2021). For the past few decades, immunotherapy has attracted tremendous research interest for its specificity, capacity to clear microscopic lesions and relapse reduction. Boosting immunity and eliminating tumors through the immune system has been the direction of all oncology researchers' efforts. Tumor-associated antigens (TAA) and tumor-specific antigens (TSA) are delivered to immune cells to trigger the specific immune response against tumor cells, thus providing a theoretical basis for tumor immunotherapy (Chen and Mellman, 2013). Currently, immunotherapies commonly employed in clinical practice include immune checkpoint inhibitors and adoptive cell therapy (ACT). Immune checkpoint inhibitors mainly include antibodies specifically targeting cytolytic T lymphocyte-associated antigen 4 (CTLA4), programmed death protein 1 (PD-1) and programmed death ligand 1 (PD-L1) (Krummel and Allison, 1995). ACT is a tumor-fighting method by introducing *ex vivo* modified immune cells into the patients. NB-associated immunotherapies mainly involved in cytokines, dendritic cell vaccines, anti-GD2 antibodies, and allogeneic hematopoietic stem cell transplantation (Sait and Modak, 2017).

Nanomaterials are known to stabilize antibodies and immune factors as well as increase the enrichment of immune factors at tumor regions, improve their targeting and effectiveness, and reduce the adverse effects associated with immunotherapy. While cancer vaccines are used to treat tumors by activating the body’s immune response with TAA, TSA and immune factors, the assembly of nanomaterials protects immune components from the internal environment and automatically target tumor-specific T cells to activate a specific immune response (van der Burg et al., 2016). Recently, TSA-containing nanovaccines were designed to produce more tumor-specific cytotoxic T lymphocytes and stronger immune responses *in vivo* compared to vaccines without nanomaterials (Kuai et al., 2017). Moreover, the combination of this vaccine with immune checkpoint inhibitors exhibited a synergistic suppressive effect on tumor recurrence (Kuai et al., 2017). While conventional ACT requires isolation of immune cells from the organism, nanoscaffolds are able to recruit and enrich immune cells *in vivo*. Poelaert et al. developed an immune cell chemotactic agent CC motif chemokine ligand 21 (CCL21) from injectable, sustained-release and optimally loaded alginate nanoformulation, which significantly prolonged the survival, reduced tumor growth and improved immune and therapeutic efficacy compared with CCL21 alone (Poelaert et al., 2020).

**FIGURE 6:**
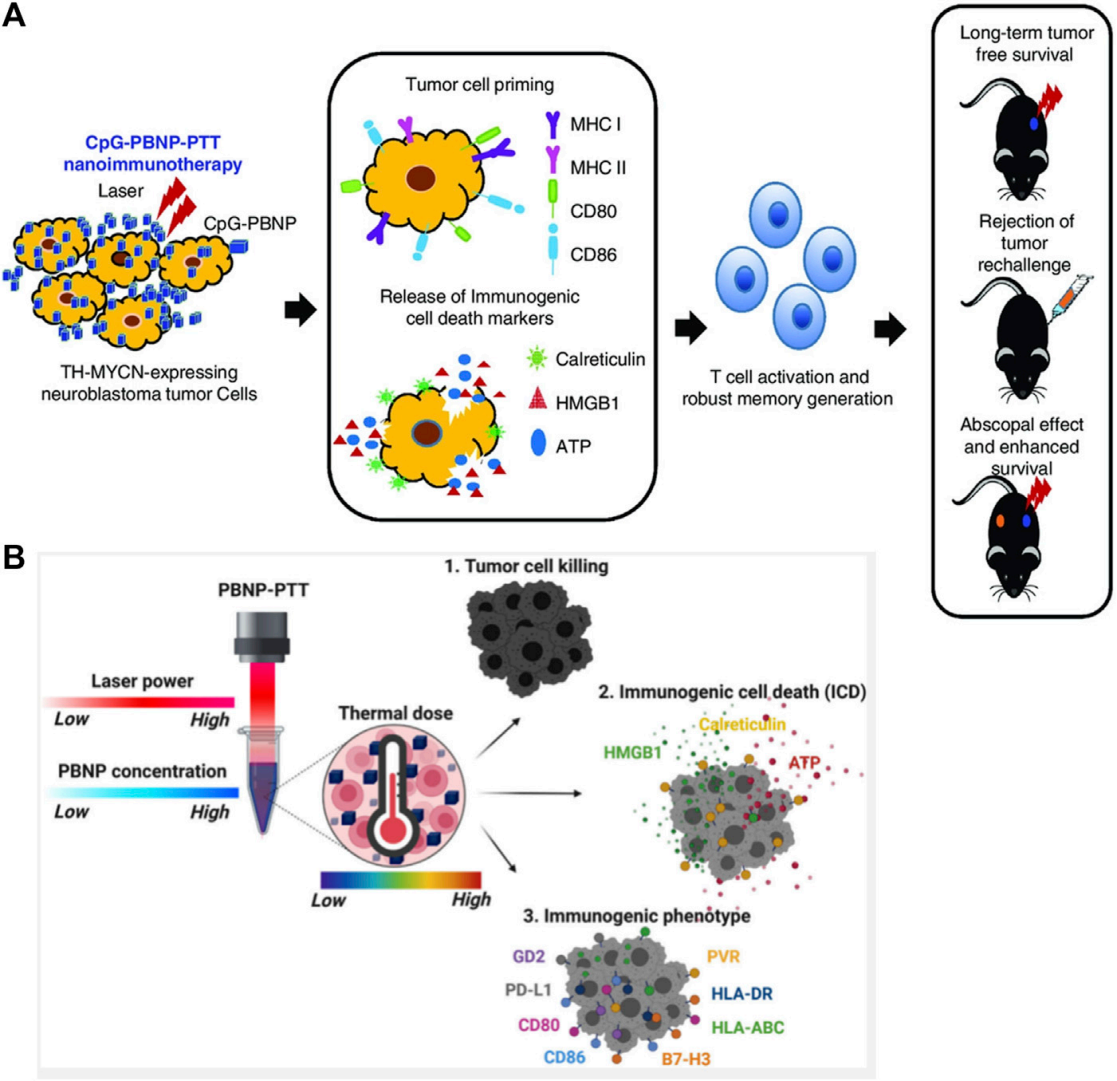
Nanotechnology for immunotherapy in NB. **(A)** Schematic of the mechanism of action of the CpG-PBNP-PTT-based nanoimmunotherapy in the TH-MYCN model of NB. Reproduced with permission from (Shukla et al., 2021). Copyright 2021, Wiley-VCH Verlag GmbH & Co. KGaA, Weinheim. **(B)** Diagram of the induction of NB cell death and immunogenic cell death (ICD) by combined PBNP-PTT therapy. Reproduced from (Sekhri et al., 2022).

Scientifically advance studies have confirmed that individual immune status is inevitably affected by multiple therapeutic options while exerting therapeutic effects. For this reason, the combination of multimodal therapies with immunotherapy is emerging as a feasible treatment option for NB. Undoubtedly, the development of nano-agents enriches these therapeutic strategies. For example, radiotherapy-induced release of inflammatory factors and TAA from *in situ* tumors will trigger tumor-specific immune responses. Thus, radiotherapy and immunotherapy may synergistically exert systemic antitumor effect (Kwong et al., 2011). Nanoparticles capturing TSA have been reported to strengthen the effect of radiotherapy and their abscopal effect while combining with radiotherapy. Juliana et al. demonstrated that combination of PTT with nanomaterials containing immune adjuvant and CTLA-4 monoclonal antibody improved T-cell levels and inhibited tumor growth in NB mice, while exerting a memory effect to suppress tumor recurrence, with superior therapeutic efficacy than CTLA-4 monoclonal antibody or PTT therapy alone (Cano-Mejia et al., 2017). They showed in their mouse model of TH-MYCN gene-driven malignant NB that PTT therapy with PBNP coated with the immune adjuvant CpG oligonucleotide (CpG-PBNP-PTT) was effective in regressing mouse tumors, improving survival time and activating t cell-mediated systemic immune responses (Figure 6A) (Shukla et al., 2021). Similarly, combining CpG-PBNP with anti-CTLA-4 immunotherapy can not only cause ablative cell death, but also alter the surface levels of co-stimulatory, antigen-presenting, and co-inhibitory molecules on NB cells (Cano-Mejia et al., 2020). Mechanistically, they elucidated that a series of thermal doses administered to NB cells using Prussian blue PBNP-based PTT (PBNP-PT) upregulated immunogenicity-related markers and enhanced the toxic killing effect of T cells on NB cells (Figure 6B) (Sekhri et al., 2022). The organic combination of nanotechnology-based therapies with immunotherapy will fully engage the complementary advantages of multiple therapies.

### Nanotechnology and Gene Therapy

Gene therapy as a novel oncology treatment has become a research hotspot in biomedicine. Currently, one of the serious challenges in gene therapy research is the issue of vehicle system (Whittle et al., 2017). Transfer vehicles applied for gene therapy include antisense nucleic acids, cationic polymers, plasmid DNA and recombinant viral vectors (Roy et al., 2005; Ullah et al., 2021). However, antisense nucleic acids and plasmid DNA are inefficiently transferred and susceptible to degradation by nucleases, while viral vectors are defective in terms of safety and immunogenicity. Therefore, it seems crucial to develop new safe and efficient gene therapy vector systems. In addition, the target gene involved in the gene therapy should ideally be tumor cell-specific, otherwise some toxic effects or other concerns can be inducted. With the rapid development of nanobiotechnology, the emergence of a new non-viral vector system, the nanogene vector system, has brought new vitality to gene therapy in NB. It has the advantages of lower immunogenicity, larger capacity, better protection of DNA fragments, higher transfection rate and lower cytotoxicity compared to viral vectors (Roy et al., 2005).

Using nanotechnology not only improves transfection efficiency, but also confers properties that make the vector more suitable for transporting exogenous genes, such as resistance to lysosomal enzymes and DNAases. In addition, it features the advantages of being easy to produce and inexpensive. Integration of nanobiotechnology and gene therapy manifests favorable application prospects. Yoshida S et al. used antisense oligonucleotides (ASO) and superparamagnetic iron oxide (SPIO) NPs as the delivery vehicles for the transcriptional regulator MAX dimeric protein 3 (MXD3) (Yoshida et al., 2020). Under the treatment of MXD3 ASO-SPIO NP complex, MXD3 expression was down-regulated and apoptosis was significantly induced in NB cells. Miguel et al. identified D-lysine polymer as an effective gene delivery vehicle and could be used as a synthetic cell-penetrating peptide for gene therapy in the SH-SY5Y cell (Sanchez-Martos et al., 2021). Another study found that rabies virus glycoprotein peptide-modified poly D, L-lactide-co-glycolide NPs could specifically target NB *in vitro* and *in vivo* and significantly inhibited tumor growth in a mouse model (Lee et al., 2016b). In addition, a novel pH-sensitive liposomal nanocarrier quatsomes (QS) platform has been reported to deliver RNA *in vivo* and *in vitro* (Boloix et al., 2022). QS-miRNA complexes are well tolerated and enable subcutaneous NB xenografts and have a potential for the treatment of high-risk NB or other cancers.

### Nanotechnology and Induction of Differentiation

Differentiation therapy aims to trigger an irreversible mature transformation of cancer cell phenotype with minimal cellular damage and to promote maturation of cancer cells, and is an alternative to conventional chemotherapy and radiotherapy as an important anti-cancer option (Yan and Liu, 2016; de Thé, 2018). Clinical observations have shown that spontaneous regression or differentiation occurs in a few cases of NB tissues (even with MYCN expression) without systemic treatment (Nakagawara et al., 2018). Therefore, it is believed that induction of mature differentiation and reversal of the malignant phenotype of tumor cells may have more favorable predispositions for NB than killing the tumor cells directly.

Studies have found that differentiation-induced substances for NB include 13-cis-retinoic acid (RA), γ-interferon, dibutyl cyclic adenosine monophosphate, sodium phenylacetate, insulin-like growth factor, nerve growth factor and glial cell-derived neurotrophic factor *in vitro*, among which RA is the most studied (Reynolds et al., 1991). The differentiation of tumor cells into normal tissue cells is modulated by genetic factors, nutritional conditions and chemical substances. However, regular administration of RA may produce toxic effects such as liver toxicity, skin chafing, and gastrointestinal damage (Cañón et al., 2004). With the in-depth research on nanomedicines, nanomaterials are able to increase the stability of tumor differentiation-inducing therapeutics, improve their targeting and ability to induce cell differentiation, and reduce the adverse effects associated with differentiation therapy.

**FIGURE 7:**
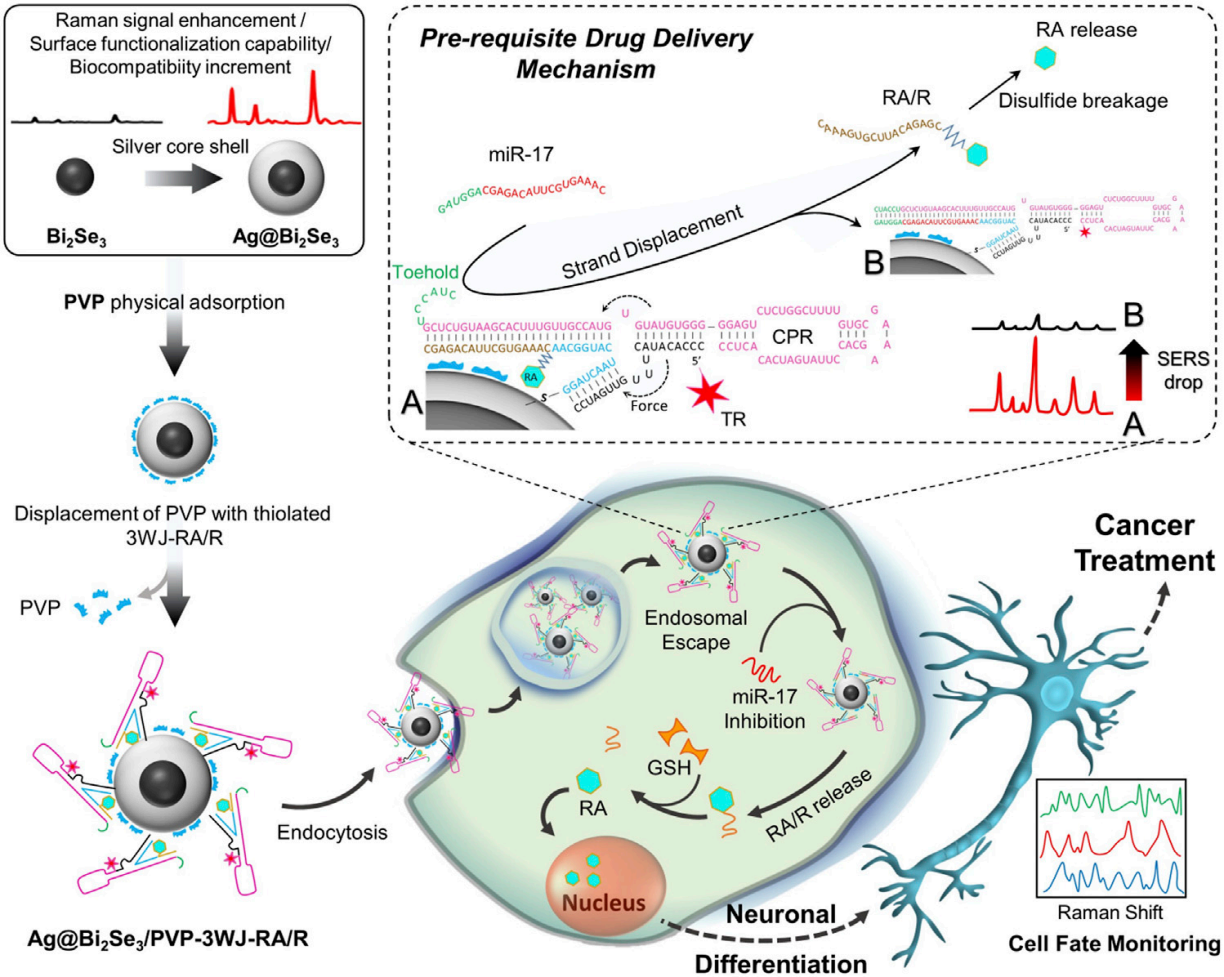
Nanotechnology for induction of differentiation in NB. Schematic diagram of the Ag@Bi2Se3/PVP-3WJ-RA/R fabrication and its application in NB cell differentiation. Reproduced with permission from: Mohsen Mohammadniaei, Jinho Yoon, Hye Kyu Choi, Virginie Placide, Bapurao Gangaram Bharate, Taek Lee and Jeong-Woo Choi. Multifunctional Nanobiohybrid Material Composed of Ag@Bi(2)Se(3)/RNA Three-Way Junction/miRNA/Retinoic Acid for Neuroblastoma Differentiation. ACS applied materials & interfaces, 2019, 11(9): 8779–8788 (Mohammadniaei et al., 2019). Copyright 2019, American Chemical Society.

The restoration of normal function or differentiated phenotype of tumor cells is associated with tumor suppressor function. It has been shown that silver NPs (AgNPs) induce neuronal differentiation by modulating reactive oxygen species, phosphatase and kinase signaling pathways in NB cell (Abdal Dayem et al., 2018). Furthermore, AgNPs-coated carriers serve as excellent drug targeting agents, and significantly promote neurite outgrowth (Abdal Dayem et al., 2018). Another study showed that graphene and graphene-associated nanomaterials exhibit biocompatibility with various cell lines and can be used as scaffolding agents to sustain cell attachment and induce proliferation and differentiation. Graphene oxide significantly promotes the differentiation efficiency of RA on NB cell by enhancing the expression of microtubule-associated protein 2 (Yeasmin et al., 2017). In a recent study, nanocomposite made of core-shelled topological insulator bismuth selenide NPs (Bi2Se3 NP) with silver (Ag@Bi2Se3) has been found to exhibit superior biocompatibility and plasmonic properties versus Ag NPs alone (Figure 7) (Mohammadniaei et al., 2019). Furthermore, they innovatively linked Ag@Bi2Se3 to cell-permeable RNA and fluoro-label with a newly developed RNA three-way junction (3WJ) structure to guide the gradual release of RA within the cell membrane. Remarkably, this designed nano-biohybrid material overcomes the long-plagued hydrophobic challenge of RA by conjugating RA to RNA strands (RA/R), while showing potent suppression of NB cell growth through differentiation induction. Together, these studies suggest that nanomaterial-based technologies may benefit the differentiation of NB cells through regulating intracellular oxidative homeostasis, activation of kinase signaling pathways, and solubility of differentiation inducers, thus potentially providing a new perspective for future NB therapy.

### Nanotechnology and Tumor Extracellular Matrix Remodeling (ECM)

The tumor microenvironment (TME) represents the internal and external environment surrounding the tumor cells, which includes not only diverse functional cells but also some supportive noncellular components such as secreted signaling molecules and ECM (Ho et al., 2020; Ganguly et al., 2022; Saw et al., 2022). As an essential component of TME, the ECM is known to be a dense network with structural proteins, bridging proteins, proteoglycans and enzymes that mainly provide biochemical and structural support for tumorigenesis and progression (Ho et al., 2020; Gonzalez-Molina et al., 2022; Jiang et al., 2022). Various chemokines, inflammatory factors, cytokines and growth factors secreted from TME-associated inflammatory cells or stromal cells collectively constitute a dynamic microhabitat possessing diverse functional states through crosstalk with the ECM (Wohlrab et al., 2014; Zhou et al., 2021). The aberrantly overexpressed ECM components in TME appear to be associated with stronger pro-invasive, pro-metastatic and pro-proliferative capacities of NB. Besides, an overly dense ECM tends to form a natural barrier that prevents cancer cells from accessing chemotherapeutics and immune cells, thus limiting the efficacy of endogenous and exogenous interference approaches against tumors to some extent (Burgos-Panadero et al., 2021). Given the oncogenic effect of abnormally high ECM expression, disturbing the interaction between ECM components and tumor cells becomes a powerful and reliable strategy for NB. However, most of the ECM-based drugs, including hyaluronidase, collagenase and proteolytic enzymes, are not applicable for oncology treatment, mainly due to their adverse effects such as short serum half-life, loss of function and insufficient accumulation in tumor area (Burgos-Panadero et al., 2021). The combination therapy of NPs with such drugs has emerged as one of the effective solutions to these problems. The matrix glycoprotein vitronectin contributes to the metastatic progression of tumors as an important mediator of crosstalk between ECM and tumor cells (Burgos-Panadero et al., 2019). Based on this, a high-affinity cyclic pentapeptide αv integrin antagonist, cilengitide, was employed to specifically target vitronectin for ECM disruption, and its combination with etoposide-loaded NPs more effectively enhanced the cytokilling effect on high-risk NB cell lines (Burgos-Panadero et al., 2021). Although nanomaterial-based ECM-targeting therapy has not been widely reported to be used for the diagnosis or treatment of NB, increasing research findings indicate that it will be one of the key directions for future research.

## Development and Application of Nanoformulations for Several Promising Therapeutic Targets in Neuroblastoma

Tumor-targeted therapy refers to the means of selectively killing tumor cells through targeting specific sites or targets of tumors without harming normal tissues, and is classified into organ-targeted therapy and molecular-targeted therapy (Rössler et al., 2008). The former mainly involves the treatments for certain diseased organs, such as targeted aggregation radiotherapy. The latter refers to the development of therapeutic agents targeting some identified carcinogenic targets such as protein molecules or gene fragments in tumor cells, such as HER2, EGFR, and GD2 (Tian et al., 2015; Tian et al., 2017; Anderson et al., 2022). Carriers are essential components for achieving targeted therapy through improving the metabolic kinetic properties of drugs, increasing drug enrichment within tumor cells and tissues, enhancing therapeutic efficacy and minimizing unwanted side effects (Markovsky et al., 2017). Commonly utilized drug-assisted carrier systems include macromolecular delivery systems, particulate delivery systems, magnetically guided formulations and multi-targeting vectors (Jiao et al., 2011). Nanomedicine vectors allow to reduce or eliminate the effects of acids, bases, salts and other biochemical factors in body fluids on the drug encapsulated in the vector and prevent the drug from being completely metabolized before reaching the target site. In addition, nanomedicine carriers with large surface area and high interfacial activity avoid recognition and phagocytosis by the immune system, thus overcoming the “biological barrier” and enabling targeted drug delivery *in vivo* (Jiao et al., 2011). Notably, nanocarriers for drug delivery are required to be both readily absorbable and degradable in the biological environment and be biocompatible, non-cytotoxic and stable in the bloodstream (Aleassa et al., 2015). Since the research on NB targets-based nanodrug has been well reported, it is necessary to summarize them accordingly (Table 3).

**TABLE 3:** Summary of nanocomposite applications of potential targets available for NB diagnosis or therapy.

Target	Function	Formulations	Nanocarrier	Observed effects	Assays	Ref.
GD2	Promoted neural differentiation, repair, invasion and immunosuppression.	IGD-Targeted	DNA nanomedicine	Selective delivery of Dox to GD2-positive NB tumor cells	*In vitro* and *in vivo*	Zhang et al. (2021a)
		Self-assembly of aptamers DB99 and MYCN-siRNA and Dox	DNA nanomedicine	Specifically Knockdown of MYCN and release of Dox in GD2-positive NB cells	*In vitro* and *in vivo*	Zhang et al. (2021b)
		Gold NPs conjugated to anti-GD2 antibody HGNPs,	Gold NPs	Enhanced both CT imaging and NK cell-mediated cancer cell killing	*In vitro*	Jiao et al. (2016)
MYCN	Promoted tumorigenesis and malignant progression of NB	Folate-nanoliposome entrapped MYCN siRNA	Folate-nanoliposome	Pro-apoptotic effect	*In vitro*	Zhu et al. (2013)
ALK	Induced cellular overproliferation and the development of NB	TL-ALK-siRNA	Anti-GD2-targeted liposomes	Antitumor activity	*In vitro* and *in vivo*	Di Paolo et al. (2011)
VEGF	Angiogenesis and tumorigenesis of NB	SiO2@LDH-Bev-DOX	SiO2@LDH-Bev NPs	Antitumor and anti-angiogenesis efficiency	*In vitro* and *in vivo*	Zhu et al. (2017)
NCAM	Mediated tumor cell metastasis	PG-NTP-PTX-PEG	Dendritic polyglycerol	Inhibited the migration of proliferating endothelial cells	*In vitro* and *in vivo*	Vossen et al. (2018)
		PGA-PTX-NTP	Polyglutamic acid	Inhibited tumor growth	*In vitro* and *in vivo*	Markovsky et al. (2017)

GD2, a dialdehyde glycoside, is poorly expressed in tissues such as the cerebellum and peripheral nerves whereas abundantly expressed in neuroendocrine system-derived tumors, rendering monoclonal antibodies designed against GD2 an effective strategy for the diagnosis and treatment of multiple neurological tumors (Sait and Modak, 2017; Pastorino et al., 2019; Rodríguez-Nogales et al., 2019). Notably, GD2 is expressed on the cell surface of almost all types of primary NB, thus it seems to be one of the most desirable targets for NB-targeted therapy (Schulz et al., 1984; Matthay, 2018). Furthermore, unlike other tumor antigens, GD2 expression is maintained on the surface of NB cell even during treatment, which provides another strong evidence for its targetability (Mount et al., 2018). Nevertheless, GD2 is also found to be expressed in certain normal tissue cells such as peripheral neurons and CNS neurons, which causes some difficulties for anti-GD2 therapy (Yu et al., 2020). Currently, the GD2 monoclonal antibodies are unable to distinguish GD2-expressing normal cells from tumor cells, which would cause indiscriminate damage to both tumor and normal cells. Therefore, the exploration of new strategies for targeting GD2 exclusively in NB cells will be the primary issue to improve the safety and efficacy of anti-GD2 therapy. Zhang L et al. constructed IGD-Target, a PH-sensitive switchable drug delivery system based on GD2 inducers and i-motif elements, was able to specifically target and arrest the growth of GD2-expressing tumor cells without affecting surrounding normal cells. This formulation greatly enhanced the targetability for GD2 and reduced the adverse effects of anti-GD2 therapy (Zhang et al., 2021a). Moreover, they have developed a GD2 aptamer (DB99)-mediated multifunctional nanomedicine (ANM) with high efficiency, precision and biocompatibility for chemotherapy and gene therapy in NB (Zhang et al., 2021b). ANM is composed of synthetic aptamer DB99 and NB-specific MYCN-siRNA reloaded with the chemotherapeutic agent DOX for intracellular delivery and release of DOX (Zhang et al., 2021b). ANM was proven to selectively repress tumor cell growth with fewer side effects on normal tissues, greatly prolonging survival by specifically targeting GD2-positive tumor sites. Given the ubiquitously expression of GD2 in NB cells, nanodiagnostic enhancers based on GD2 have also been exploited for NB imaging. The gold NPs (GNPs) conjugated with tumor-targeting anti-GD2 antibodies, or HGNPs can specifically augment CT imaging while simultaneously stimulate NK cell-mediated killing of NB cells, which exerts a dual role of activating innate cytoimmune responses and potentiating imaging (Jiao et al., 2016). All of these suggest that GD2 aptamer-mediated targeted drug delivery systems will have promising applications in the precise treatment of NB.

MYCN gene amplification occurs in approximately 20–25% of NB patients and is typically associated with a poor prognosis for high-risk disease (Floros et al., 2021; Putra et al., 2021). Although MYCN performs critical role in the malignant phenotype of NB cells, it is extremely difficult to be targeted directly (Otto et al., 2009; Braoudaki et al., 2021). Current treatments for MYCN amplification are mainly indirect targeting strategies involve in manipulating its transcription, translation, protein stability and target gene transcription (Rickman et al., 2018; Ommer et al., 2020; Wang et al., 2022b). However, the development and application of nanomaterials for MYCN in NB therapy remains largely unknown. Based on the overexpression of folate receptors on the surface of neuroblastoma cells, folate nanoliposome delivery system, a low-toxic and high specificity nanomaterial, was utilized for encapsulating MYCN-siRNA to achieve MYCN-specific interference in tumor tissues and promotion of cell apoptosis (Zhu et al., 2013).

Vascular endothelial growth factor (VEGF), a heparin-binding growth factor specific for vascular endothelium, is an essential factor in angiogenesis *in vivo* (Rodríguez-Nogales et al., 2019). The application of nanoformulations targeted to VEGF for NB therapy is also not an inappropriate therapeutic option. Indeed, selective binding of bevacizumab to VEGF proteins coupled with SiO2-layered dihydroxane DOX-loaded nanocomposites for NB treatment significantly improved DOX cellular uptake and targeting delivery efficiency, inhibited tumor cell angiogenesis, reduced side effects of DOX, and inhibited VEGF-mediated angiogenesis and tumorigenesis (Zhu et al., 2017).

Neural cell adhesion molecule (NCAM) overexpressed in tumor initiating cells and tumor endothelial cells is also considered as a meaningful therapeutic target for NB (Wachowiak et al., 2008; Vossen et al., 2018). A new polyglutamic acid-PTX-NCAM Targeting Peptide (PGA-PTX-NTP) conjugate was developed and evaluated experimentally in neuroblastoma *in vivo*, and showed improvement in the NCAM-targeted tumor cell killing rate, prolonged drug action time and reduction of toxic effects (Kiselyov et al., 2009; Markovsky et al., 2017). Subsequently, a PG conjugate of polyethylene glycolated NCAM-targeted dendritic polyglycerol (PG) with PTX and NCAM-targeted peptide (NTP) (PG-NTP-PTX-PEG) was further developed to effectively suppress tumor angiogenesis (Vossen et al., 2018).

Anaplastic lymphoma kinase (ALK) has been identified as an oncogenic driver in several cancers especially in NB and is considered a critical contributor for tumorigenesis and a promising therapeutic target of NB (Frentzel et al., 2017; Li et al., 2021a). It has been reported that the novel ALK inhibitor X-396 in combination with an ALK-siRNA carrying targeted liposomes (TL-ALK-siRNA) exhibited superior drug bioavailability, moderate half-life, elevated plasma concentrations, and significantly prolonged lifespan in NB mice regardless of ALK gene mutation status (Di Paolo et al., 2011). More researches on new targets and corresponding potential nanocarriers are expected to further promote the advancement of NB targeted agents in the future.

## Biocompatibility and Safety of Nanomaterials

Although numerous studies have confirmed that the emerging of nanomaterials has indeed facilitated the targeted diagnosis and treatment of various tumors, it is not easy to give a definite answer to the ambitious issue of biocompatibility and safety of nanomaterials. In this section, we will discuss the opportunities and challenges of nanomaterials, which are highly utilized and have achieved some breakthroughs in NB diagnosis and therapy, such as Gold NPs and AgNPs.

Noble metal NPs have attracted a lot of interest in cancer research owing to their unique optical properties and good biocompatibility (Zhao et al., 2022). Combining noble metal-based nanotechnology with diagnostic methods and therapies (from traditional radiotherapy to emerging immunotherapy) has improved the accuracy and efficiency of cancer diagnosis and treatment (Jiao et al., 2016; Mohammadniaei et al., 2019; Zhan et al., 2021). Gold NPs are the most frequently utilized noble metal NPs in tumor diagnosis and treatment due to their unique radiosensitizing properties, good biocompatibility, and relatively low toxicity (Boisselier and Astruc, 2009; Kovács et al., 2022; Li et al., 2022). Studies have also shown that GNPs may possess certain targeting properties, as demonstrated by the fact that GNPs selectively act on heparin-binding proteins, such as EGFR and VEGFR-2 (Mukherjee et al., 2005; Huang et al., 2021; Zhao et al., 2022). In addition, it improves the precise delivery and bioavailability of drugs by covalently and non-covalently binding and transporting drug molecules (Kim et al., 2012; Ren et al., 2021). Currently, numerous *in vitro* and *ex vivo* studies have almost demonstrated that the core components of AuNP do not possess significant biological toxicity, but their coating stabilizers, such as cetyltrimethylammonium bromide (CTAB), may present a risk of toxic effects on the organism (Li et al., 2014; Zhao et al., 2022). Moreover, the inherent physical properties of AuNPs also affect their safety, such as particle size and oxidation state (Li et al., 2014; Zhao et al., 2022). It is generally believed that the core of AuNPs larger than 5 nm behaves more inert, while the surface of AuNPs smaller than 2 nm shows an unusual chemical reactivity. For example, AuNPs with a diameter of 1.4 nm produced significant cytotoxicity by inducing oxidative stress and mitochondrial damage (Pan et al., 2009). Toxicity studies of citrate-capped AuNPs in mice by Chen et al. showed that small (3–5 nm) and large AuNPs (30 and 100 nm) were not toxic, while medium-sized AuNPs (8, 12, 17 and 37 nm) caused severe toxic effects (Chen et al., 2009). The high exposure of surface areas of anisotropic AuNPs poses a more serious toxicity risk due to oxidation than anisotropic AuNPs (Zhao et al., 2022). Therefore, it remains doubtful whether the results of *in vitro* cellular experiments could be translated equally to *in vivo* and clinical applications in the future. Initially silver NPs were used as antimicrobial agents due to their cytotoxicity (Panáček et al., 2018). It was subsequently proved to kill tumor cells through inducing cellular oxidative stress and affecting mitochondrial membrane stability in vivo and *in vitro* experiments (Cao et al., 2022; Ren et al., 2022; Skóra et al., 2022). As a result, silver nanomaterials have become a hot research topic in tumor therapy. However, after all, silver ions are highly cytotoxic as heavy metals and are prone to undesired toxic effects (Choudhary et al., 2022). Moreover, similar to gold nanoparticles, the size and oxidation properties of silver nanoparticles also determine their cytotoxicity (Soenen et al., 2015).

Although, the application of NPs has effectively overcome the shortcomings of conventional oncology diagnosis and therapy, nanomaterial-based cancer treatment still faces many challenges. For example, when contrast agents in lipid therapeutic nanomedicines interact with biological materials due to their incompatibility or potential toxicity, adverse reactions such as inflammation, immune response or related diseases in the organism will occur (Dilnawaz et al., 2018; Bukhari et al., 2021). Superparamagnetic Fe3O4 NPs were once considered to be relatively inert carriers for therapeutic and diagnostic drugs. However, one study found that intravenous injection of Fe3O4 NPs induced inflammatory responses, cytotoxic damage and respiratory toxicity in mice (Hurbankova et al., 2017). Encouragingly, the biosafety of PBNPs has been evaluated in normal and cancer cell lines as well as in animal models (Busquets and Estelrich, 2020). It has been demonstrated that PBNPs do not cause significant organ damage or abnormalities in heart, liver, spleen, lungs and kidneys in mice (Fu et al., 2014; Zhao et al., 2018). Importantly, PBNPs exhibit good biocompatibility in humans, as demonstrated by their long-term presence in human serum without causing significant toxicity (Shokouhimehr et al., 2010; Montiel Schneider et al., 2018). Most current studies have focused only on the efficacy of nanomaterials *in vitro* and *in vivo*, while the observation of their resulting toxic effects is often neglected. In-depth study of their toxicological mechanisms is essential. The current NPs should be continuously improved accordingly based on the mechanism, so as to maximize their effectiveness and minimize the toxic effects.

## Conclusion and Future Directions

NB is a highly heterogeneous tumor with multiple non-specific clinical manifestations, and its prognosis is determined by multiple factors such as the patients' age and histological and biological characteristics of the tumor. Despite the growing maturity of the current NB diagnostic technology combined with various approaches, it still needs to be improved in terms of enhancing the diagnostic accuracy and simplifying the diagnostic method. Therapies available for NB have been developed, ranging from traditional chemoradiotherapy to the latest advances such as immunotherapy and gene therapy. While optimization of these treatments has improved the prognosis and outcome for the majority of patients, it remains incurable in approximately half of cases with high-risk NB.

In this review, we summarized the development and application of nanoformulations based on the existing potential therapeutic or diagnostic targets of NB. As most of the results are obtained from the preclinical study, there is still a lack of sufficient clinical trials for validation. Excitingly, nanotechnology has been widely studied in the field of NB diagnosis and treatment due to its excellent performance in many aspects, thus presenting an opportunity to resolve the difficulties in the traditional diagnosis and treatment of NB. Yet, as an emerging technology, nanotechnology remains to be further optimized to accommodate current clinical needs in terms of the shortcomings in material preparation, biosafety, toxicity, analytical methods and mechanical investigations. Furthermore, nanomedicine-based chemotherapy integrated with other therapeutic modalities such as PDT and immunotherapy have shown synergistically enhanced anti-cancer effects. In addition to traditional therapeutic approaches, novel approaches focusing on the suppression of NB cell growth and malignant phenotype based on programmed cell death are ongoing directions in nanomedicine, such as ferroptosis induction (Zafar et al., 2021). Furthermore, some researchers achieved drug loading and transport by effectively utilizing the physiological conditions of the organism itself as biocompatible carriers for the purpose of certain tumor treatment, such as red blood cells (RBCs) (Li et al., 2021b; Wu et al., 2021). Most of the previous studies on the application of nanomaterials in NB diagnostics and therapeutics are mainly conducted through cellular and animal experiments, and more efforts are required to accomplish the clinical translation of these scientific findings. We believe that the advancement of nanomedicine will definitely provide an unprecedented opportunity for clinically precise treatment of NB patients in the future, such as tumor-specific targeting, favorable biocompatibility and an ease in functionalization. More importantly, a number of nanomedicines have been approved for clinical trials or launched for clinical application. It is believed that nanomedicine will bring a brighter perspective for the diagnosis and therapy of NB with a rapid and enormous advancement in the coming future.
